# Colo-cutaneous fistula: an unusual complication after hemilaminectomy and microdiscectomy

**DOI:** 10.1093/jscr/rjaf167

**Published:** 2025-03-27

**Authors:** Isabella Zappala, Joseph Do Woong Choi, Bishoy Soliman, Nimalan Pathma-Nathan

**Affiliations:** Department of Surgery, Westmead Hospital, Cnr of Hawkesbury and Darcy Roads Westmead, Sydney 2145, Australia; Department of Surgery, Westmead Hospital, Cnr of Hawkesbury and Darcy Roads Westmead, Sydney 2145, Australia; Faculty of Medicine and Health, The University of Sydney, Camperdown NSW 2050, Sydney, Australia; Department of Surgery, Westmead Hospital, Cnr of Hawkesbury and Darcy Roads Westmead, Sydney 2145, Australia; Department of Surgery, Westmead Hospital, Cnr of Hawkesbury and Darcy Roads Westmead, Sydney 2145, Australia; Faculty of Medicine and Health, The University of Sydney, Camperdown NSW 2050, Sydney, Australia

**Keywords:** colocutaneous fistula, microdiscectomy, hemicolectomy, chronic wound infection

## Abstract

Although the current literature points to surgical complication as the most common cause of entero-cutaneous fistulae, the formation of a colo-cutaneous fistula following a microdiscectomy is an unusual complication. Derivation of iatrogenic fistulae is rare, however, can carry increased rates of patient mortality and may require definitive surgical intervention. The authors present a clinical manifestation of a colo-cutaneous fistula between the descending colon and lumbosacral back wound from a previous microdiscectomy.

## Introduction

Colo-cutaneous fistulae are abnormal connections between the colonic mucosa and the skin epithelium. Based on the anatomical location, colonic fistulae produce low output volumes however present with significant rates of morbidity and mortality leading to prolonged hospitalisations, nutrition and electrolyte optimisation and sepsis control [1]. The vast majority of colo-cutaneous fistulae are formed following iatrogenic complications in surgery and relate to patient- and technique-specific factors [2]. Accounts of colo-cutaneous fistulae formation have emerged from missed enterotomies, leaking anastomoses following bowel resection and erosion of surgical mesh following hernia repair [3]. To the author’s knowledge, there have been no accounts of colo-cutaneous fistula formation following elective microdiscectomy.

## Case report

A 37-year-old lady with a background history of chronic lower back pain and left L5 nerve compression was referred for a left hemicolectomy for management of a colo-cutaneous fistula, which developed secondary to a chronic infected lumbar wound following lumbosacral microdiscectomy.

Symptoms of lower back pain and left lower limb sciatica in the L5 cutaneous distribution commenced 2.5 years ago after an episode of heavy lifting. Failure of symptom control following medical management prompted insertion of a spinal cord stimulator 6 months later. In the same month, the patient developed a left-sided foot drop.

The patient underwent a left L4/L5 and L5/S1 hemilaminectomy and microdiscectomy for L4/L5 + L5/S1 disc protrusion in addition to spinal cord stimulator repositioning. The operation was performed by translaminar approach. There were no complications during the procedure and no concern for peritoneal breach or bowel injury. She was discharged with routine physiotherapy follow-up.

11 days post-operatively, she re-presented to an emergency department with fevers, proximal lumbar wound dehiscence and underlying fluctuance. Ultrasound showed a collection at the wound site measuring 10 × 30 × 5mm. Wound cultures returned heavy growth of normal skin flora with polymorphological cells and gram-positive cocci. She was treated with intravenous cephazolin then discharged on oral cephalexin.

A wound review was performed at post-operative day 14. Ultrasound-guided aspiration returned light growth *staphylococcus epidermidis*. She remained on cephalexin. After a week of antibiotic therapy, the integrity of the wound did not improve. The distal aspect of the wound began to dehisce prompting wound revision and washout. Cultures returned growth of methicillin-resistant *staphylococcus aureus*. She was placed on a 6-week course oral clindamycin.

Despite all interventions, the patient reported ongoing subjective fevers, debilitating back pain, left lower limb paraesthesias and foot drop. She had multiple presentations to emergency departments for analgesia, septic screens and referrals to her neurosurgeon for ongoing management. She was eventually referred to a second neurosurgeon for revision of left L4/L5 and L5/S1 microdiscectomy, where smaller residual disc bulge and granulation tissue were removed around the roots.

Over the following nine months, the patient had multiple re-presentations with ongoing lumbar wound collections measuring up to 7 cm in size. This required a series of aspirations, multiple wound washouts/debridements, application of vacuum-assisted closure (VAC) dressings and various antimicrobials during inpatient encounters. The wound eventually cultured *Klebsiella pneumoniae* and began maturing into a sinus tract. CT fistulogram demonstrated a thin track of contrast extending from the tip of the catheter through the posterior wall of the descending colon directly into the lumen of the descending colon, consistent with a colo-cutaneous fistula. A colonoscopy at this time showed no abnormalities to the colonic mucosa up to the hepatic flexure.

**Figure 1:**
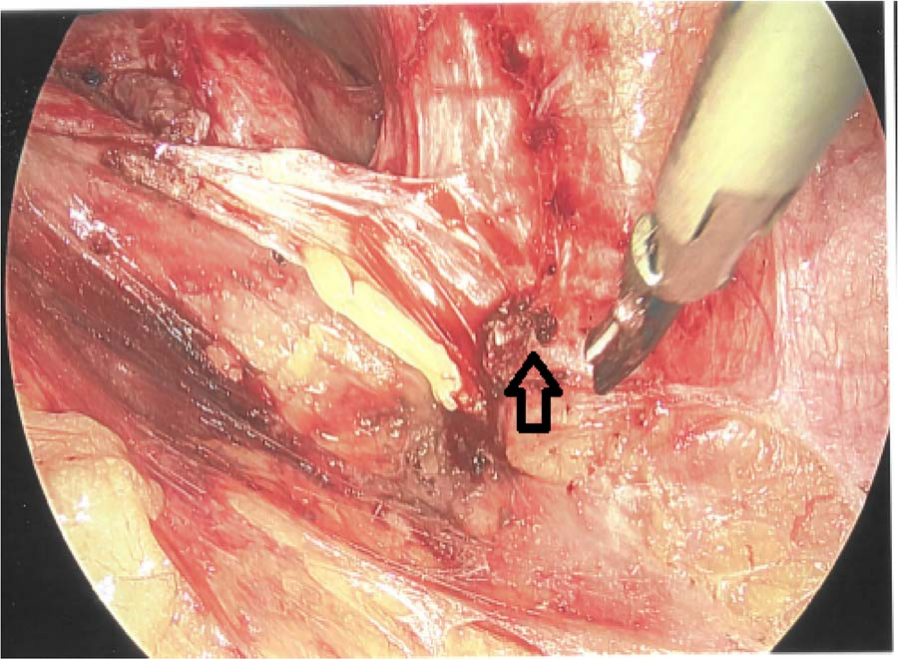
Laparoscopic view of the excised colocutaneous fistula (arrow). The descending colon has been retracted medially.

**Figure 2:**
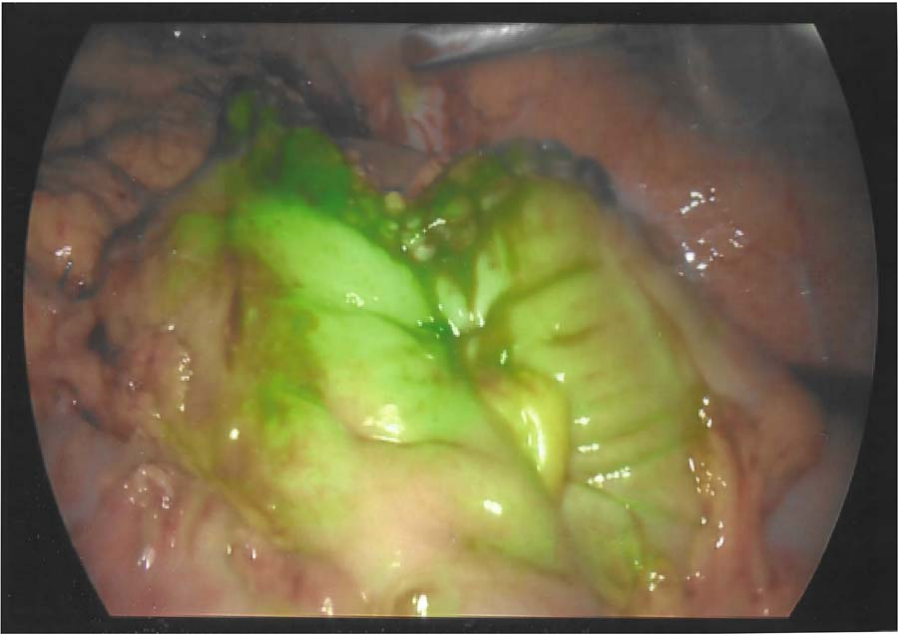
Indocyanide green (ICG) angiography demonstrating good perfusion at the colo-colonic anastomosis.

As a result, a laparoscopic left hemicolectomy was performed. The descending colon was medialised by incising the White line of Toldt from the proximal sigmoid colon, with dissection of splenocolic, phrenicocolic and the pancreaticocolic ligaments proximally. The colocutaneous fistula was identified during medialisation of the proximal descending colon and was excised (Fig. 1). There were chronic changes of inflammation and fibrosis around this region, without an abscess cavity. The distal transverse colon was then mobilised by dissecting the gastrocolic ligament to enter the lesser sac. The mobilised colon was then exteriorised through a midline laparotomy, the left colic and the left branch of the middle colic arteries were ligated and an extracorporeal side to side, functional end to end stapled anastomosis occurred using NTLC 75 mm blue reload stapler between the distal transverse and the distal descending colon. Indocyanide Green (ICG) angiography was used to confirm good perfusion at the anastomosis (Fig. 2). A well vascularised omental patch covered the remnant fistula tract on the lateral posterior abdominal wall. Postoperative recovery was uncomplicated. Histopathology was consistent with a fistula tract without evidence of malignancy.

**Figure 3:**
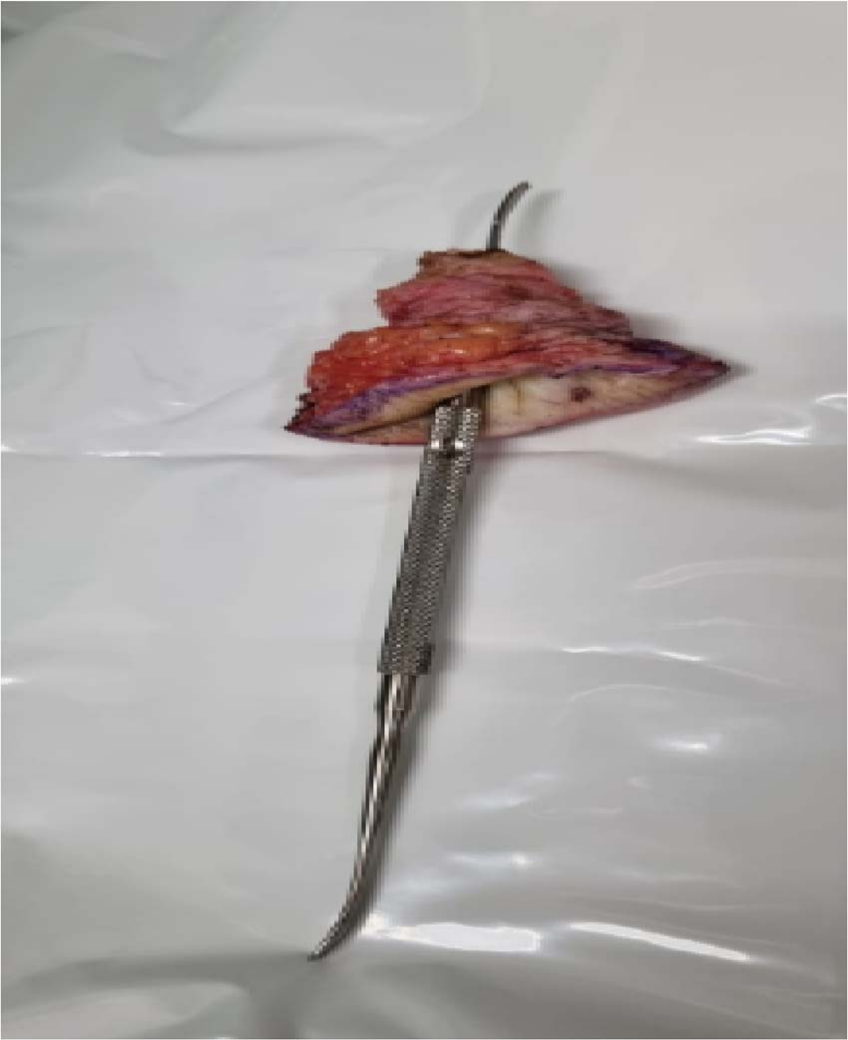
Excised back fistula with tract illustrated with probe.

The wound healed well post-operatively. The patient returned for planned excision of remaining fistula (Fig. 3) and primary closure of the skin. Her postoperative follow-up remains unremarkable with no further wound complications.

## Discussion

To the best of our knowledge, this is a novel case of a colo-cutaneous fistula after microdiscectomy and laminectomy via a translaminar approach. Only percutaneous nephrolithotomies by retroperitoneal approach have recorded rates of colo-cutaneous fistulae formation [4]. Of the 5039 procedures performed, colonic perforation complicated 15 of those procedures with 5 of the cases presenting with a colo-cutaneous fistula post-operatively. In our case, no direct damage to the colonic wall was seen, so colo-cutaneous fistula formation is rare in this setting.

Concerns for a colo-cutaneous fistula started to arise when the wound cultured *K. pneumoniae* prior to the left hemicolectomy. This led to a CT fistulogram which confirmed the unusual diagnosis. Definitive repair of a colocutaneous fistula is considered after the wound fails to heal with conservative management within 12 weeks [5].

Risk factors that contribute to colo-cutaneous fistulae formation can be divided into surgical factors and patient factors, which include additional co-morbidities, infection status, malignancy, previous radiation, bowel diseases, previous bowel trauma and foreign body exposure [6]. Our patient exhibited no risk factors therefore, fistula formation was likely to be a result of chronic wound infection after hemilaminectomy and microdiscectomy. The mechanism remains unknown; however, it may have arisen from chronic inflammation secondary to initial wound infection, with extension into the retroperitoneal space and then to the adjacent descending colon. Clinicians should maintain a high index of suspicion to investigate for a colo-cutanoeus fistula particularly after persistent wound infections after lumbosacral surgery, even though there were no concerns for retroperitoneal or peritoneal injury.

The case study is not without limitations. First, it highlighted successful management of a patient with concurrent rare pathologies that acted synergistically. The treatment pathway described was not based on extensive evidence, as it does not exist in the literature. Additionally, the authors chose not to further investigate cause of impaired wound healing, however, this may not be generalisable as younger patients with less comorbidities may not exhibit direct cause and effect relationships. Despite these limitations we have highlighted an unusual, highly-morbid presentation requiring prompt surgical management, and recommend general surgeons to maintain a broad set of differentials when undertaking lumbosacral surgery.
